# Can cases and outbreaks of norovirus in children provide an early warning of seasonal norovirus infection: an analysis of nine seasons of surveillance data in England UK

**DOI:** 10.1186/s12889-022-13771-z

**Published:** 2022-07-20

**Authors:** Anna L. Donaldson, John P. Harris, Roberto Vivancos, Sarah J. O’Brien

**Affiliations:** 1 NIHR Health Protection Research Unit in Gastrointestinal Infections, University of Liverpool, Liverpool, UK; 2 Institute of Population Health, University of Liverpool, 2nd Floor, Block F, Waterhouse Buildings, 1-5 Brownlow Street, Liverpool, L69 3GL UK; 3 Field Epidemiology Service, Public Health England, Liverpool, UK; 4 Cumbria and Lancashire Health Protection Team, Public Health England, Preston, UK

**Keywords:** Norovirus, Children, Schools, Outbreaks, Surveillance

## Abstract

**Background:**

Children are important transmitters of norovirus infection and there is evidence that laboratory reports in children increase earlier in the norovirus season than in adults. This raises the question as to whether cases and outbreaks in children could provide an early warning of seasonal norovirus before cases start increasing in older, more vulnerable age groups.

**Methods:**

This study uses weekly national surveillance data on reported outbreaks within schools, care homes and hospitals, general practice (GP) consultations for infectious intestinal disease (IID), telehealth calls for diarrhoea and/or vomiting and laboratory norovirus reports from across England, UK for nine norovirus seasons (2010/11–2018/19). Lagged correlation analysis was undertaken to identify lead or lag times between cases in children and those in adults for each surveillance dataset. A partial correlation analysis explored whether school outbreaks provided a lead time ahead of other surveillance indicators, controlling for breaks in the data due to school holidays. A breakpoint analysis was used to identify which surveillance indicator and age group provided the earliest warning of the norovirus season each year.

**Results:**

School outbreaks occurred 3-weeks before care home and hospital outbreaks, norovirus laboratory reports and NHS 111 calls for diarrhoea, and provided a 2-week lead time ahead of NHS 111 calls for vomiting. Children provided a lead time ahead of adults for norovirus laboratory reports (+ 1–2 weeks), NHS 111 calls for vomiting (+ 1 week) and NHS 111 calls for diarrhoea (+ 1 week) but occurred concurrently with adults for GP consultations. Breakpoint analysis revealed an earlier seasonal increase in cases among children compared to adults for laboratory, GP and NHS 111 data, with school outbreaks increasing earlier than other surveillance indicators in five out of nine surveillance years.

**Conclusion:**

These findings suggest that monitoring cases and outbreaks of norovirus in children could provide an early warning of seasonal norovirus infection. However, both school outbreak data and syndromic surveillance data are not norovirus specific and will also capture other causes of IID. The use of school outbreak data as an early warning indicator may be improved by enhancing sampling in community outbreaks to confirm the causative organism.

## Introduction

Norovirus is the single most common cause of infectious intestinal disease (IID) in high-income countries, accounting for approximately 11–16% of community cases [1–4]. In the UK, it affects nearly 5% of the population every year [5]. Norovirus infection occurs all year round but is more common during the winter months (December to February in the Northern Hemisphere) [6]. Norovirus typically causes a mild, self-limiting illness characterised by vomiting, watery diarrhoea, abdominal cramps and fever, with symptoms typically lasting two to three days [7]. However, the severity of disease and duration of symptoms can be affected by factors such as age and co-morbidity, with hospital patients found to experience more prolonged illness [8–10]. Norovirus is highly transmissible due to the low infectious dose and high levels of viral shedding [11], with as few as ten to one hundred particles sufficient to cause infection [12]. It can spread through faecal-oral transmission as well as being widely dispersed by vomit where it can transmit to others via inhalation, contamination of surfaces or direct contamination of hands [12, 13]. Consequently, it is a common cause of outbreaks in semi-enclosed environments, such as hospitals, nursing homes and schools [14, 15]. Each year norovirus causes widespread disruption to healthcare services and has been estimated to cost the global economy $4.2 billion in healthcare costs and $60.3 billion in societal costs per annum [16]. Each year in the UK, norovirus is estimated to cause between 6 000 and 18 000 hospital admissions, 30 000 accident and emergency attendances, 160 000 general practice (GP) consultations and 56 000 calls to telehealth services [17].

Children are thought to be important drivers of norovirus infection and experience prolonged symptoms and viral shedding, reduced immunity and higher levels of infectiousness [18–22]. Their high numbers of close social contacts, especially in home and school environments enables the spread of infection to both child and adult age groups [23, 24]. Young children have one of the highest incidence of norovirus [3, 25, 26], and household contact with a symptomatic child is a risk factor for infection in older children and adults [27–29]. Mathematical modelling has predicted paediatric norovirus vaccination could prevent 18–21 times more cases than elderly vaccination by providing both direct protection to children and indirect protection to adults [30]. In addition, there is evidence that cases in children may start increasing earlier in the norovirus season than cases in adults [25]. This raises the question as to whether cases in children could provide an early warning of seasonal norovirus before cases start increasing in older, more vulnerable age groups. This study uses national surveillance data for England, UK to explore whether cases of norovirus in children and outbreaks of IID in schools occur earlier in the season than cases and outbreaks amongst adult age groups and could, therefore, act as an early warning of seasonal norovirus.

## Methods

### Data sources

National surveillance data held by Public Health England (PHE) were requested over a 10-year period (1^st^ January 2010 to 31^st^ December 2019). Data were extracted on reported outbreaks within schools, care homes and hospitals, general practice (GP) consultations for IID, calls for diarrhoea and/or vomiting to remote telehealth services, which provide telephone-based health advice and information, and laboratory norovirus reports from across England, UK.

Outbreak surveillance of IID has been in existence in the UK since 1992 and data on outbreaks are currently collected via two reporting systems. Since 2009, hospital norovirus outbreaks have been reported nationally via the web-based Hospital Norovirus Outbreak Reporting System (HNORS), although participation and reporting are voluntary [31]. IID outbreaks in other settings are voluntarily reported to local Public Health teams, who record details of the outbreak and the subsequent management on a national web-based system [32]. An outbreak is defined as two or more cases linked in time or place, or a greater than expected rate of infection compared with the usual background rate for a given place and time [33]. Outbreaks are recorded as suspected or laboratory confirmed, depending on whether a causative organism has been isolated.

Data on GP consultations and telehealth calls form part of PHE’s National Real-time Syndromic Surveillance Service, which collects and augments data on presenting symptoms and/or suspected diagnoses from different parts of the healthcare system across England [34]. In general practice, syndromic indicators have been developed based on the Read code system, which is the recommended national diagnostic classification system for GPs [35]. These syndromic indicators include gastroenteritis, vomiting, and diarrhoea although each indicator may be triggered by a variety of different Read codes. Data on GP in-hours consultations are collected through a sentinel surveillance system which covers approximately 12% of England’s population and has been monitored by PHE since 2012. Telehealth services (NHS 111 and its predecessor NHS Direct) utilise electronic clinical algorithms, which contain a series of questions relating to a reported symptom [36]. Syndromic surveillance is based on monitoring how often these algorithms are triggered and identifying exceedances from the normal background level. Relevant algorithms for IID include both vomiting and diarrhoea. NHS Direct was in operation from 2001 until 2013, when the service was replaced by NHS 111. During the piloting and transition to NHS 111 (2012–2013), the coverage of both systems was reduced and therefore NHS 111 data was only included from the 2014/15 norovirus season onwards.

Data on positive laboratory samples are reported to PHE via two mechanisms. The statutory notification system within the UK makes it mandatory for clinicians to report suspected cases of certain infectious diseases and laboratories must inform PHE when they confirm a notifiable organism within a specimen sample [37]. Norovirus is not classed as a notifiable organism, but both suspected food poisoning and infectious bloody diarrhoea are formally notifiable. In addition, there are voluntary reporting systems established with the majority of laboratories across the country, who submit weekly electronic reports of isolated organisms, including norovirus, to Public Health England.

### Data analysis

Weekly-level data were analysed according to the norovirus seasons, with the season considered to start in calendar week 27 and end in calendar week 26 of the following year. Data were only included if they were available for the complete norovirus season. The analysis incorporated outbreak data and laboratory data from nine norovirus seasons (2010/11–2018/19), GP data from seven seasons (2012/13–2018/19) and NHS 111 data from five (2014/15–2018/19). For the analysis, cases were divided into child and adult age groups. Both NHS 111 and GP data contained pre-determined age categories, so the age boundaries for children and adults varied depending on the categories available within each dataset. For laboratory and NHS 111 data, children were defined as 0–15 years and adults ≥ 16 years. For GP data, the alternative definitions of 0–14 years and ≥ 15 years were used. Cases with missing or invalid data on age were excluded from the analysis.

A descriptive analysis was undertaken to explore the number of cases and outbreaks reported, time trends and seasonality within each dataset. Median season week and cumulative proportions were used to identify which surveillance indicator and age group were reported earliest in the norovirus season. A Spearman’s rank correlation analysis was used to compare the temporal patterns of cases in children with those in adults, and to identify any lead or lag times between the age groups for laboratory, NHS 111 and GP data. For each dataset, data were broken down into child and adult age groups and then aggregated by norovirus season week. A further correlation analysis was undertaken to explore whether school outbreaks provided a lead time ahead of other surveillance indicators. To adjust for the natural breaks in school outbreak data, a Spearman’s rank partial correlation was undertaken, controlling for school holidays. To allow data to be combined from across multiple years, school holidays were assumed to fall on the same weeks each year. The selected weeks were based on existing literature [38]. For both analyses, lead or lag time were determined by the week with the highest positive correlation up to ± 4 weeks.

Finally, a breakpoint analysis was conducted to identify which surveillance indicator and age group provided the earliest warning of the norovirus season. Each surveillance indicator was analysed as a single timeseries, spanning multiple norovirus seasons, regressed against a constant. A breakpoint represented a structural change in the regression model. A breakpoint function was applied which allowed for multiple breakpoints to be detected across the study period [39], allowing for one or more norovirus peaks to be identified in each dataset each year. No limits were put on the number of possible breakpoints across the study period. Data were smoothed prior to analysis, using a 4-week rolling average, to mitigate the effects of breaks in data due to school holidays. The minimum number of observations between breakpoints was set to 13 weeks (3 months). This was selected to account for the prolonged break in school outbreak data over the summer months and ensure breakpoints were not triggered when outbreak reporting re-commenced after school holidays. The season week of the first breakpoint in each norovirus season was extracted, alongside 95% confidence intervals (CI), to identify which surveillance indicator and age group provided the earliest warning of the norovirus peak each year. All analysis was undertaken in R 4.0.2 [40].

## Results

For the norovirus seasons 2010/11 to 2018/19, laboratory surveillance detected 65 361 cases of confirmed norovirus infection, 18% of which were in children under the age of 16 years (Table 1). Over the same time period, 33 051 IID outbreaks were reported in schools, care homes and hospitals. Care homes accounted for the largest proportion of these (57%), whilst 33% occurred in hospitals, and 10% were reported in schools. From 2012/13 to 2018/19 there were over 6 million reported GP consultations for IID and over the course of five norovirus seasons, NHS 111 received over 1.1 million calls for diarrhoea and 1.7 million calls for vomiting. Whilst children accounted for a third of GP consultations for IID, they were responsible for nearly half of all calls to NHS 111 for vomiting.

**Table 1:** Characteristics of included surveillance datasets

**Surveillance dataset** **(2010/11 – 2018/19)**	**Total reported**	**Median number of cases/outbreaks reported per norovirus season**^**a**^** (IQR)**	**Median season week of reported cases/outbreaks (IQR)**
***Outbreaks***			
Schools	3 168	344 (285 – 413)	25 (19–36)
Care homes	19 000	2 215 (1 975 – 2 391)	29 (20–38)
Hospitals	10 883	948 (732 – 1 569)	30 (23–38)
***Laboratory norovirus reports***			
Children (0-15yrs)	11 463	1 287 (1 116 – 1 449)	28 (17–39)
Adults (≥ 16yrs)	53 898	6 011 (5 308 – 7 075)	32 (24–39)
All	65 361	7 089 (6 424 – 7 912)	32 (23–39)
***NHS 111 calls for diarrhoea***^b^			
Children (0-15yrs)	470 928	95 159 (94 450 – 96 568)	28 (16–40)
Adults (≥ 16yrs)	711 480	142 355 (141 547 – 142 677)	27 (14–40)
All	1 182 408	238 923 (233 941 – 239 620)	27 (15–40)
***NHS 111 calls for vomiting***^b^			
Children (0-15yrs)	841 587	167 614 (165 405 – 171 424)	28 (17–39)
Adults (≥ 16yrs)	868 772	175 200 (170 481 – 177 051)	27 (14–39)
All	1 710 359	341 905 (340 605 – 346 398)	27 (16–39)
***GP consultations for IID***^c^			
Children (0-14yrs)	2 059 558	312 334 (231 719 – 341 421)	28 (16–39)
Adults (≥ 15yrs)	4 362 475	658 813 (518 330 – 731 522)	26 (13–39)
All	6 422 033	971 147 (750 049 – 1 072 942)	27 (14–39)

^a^ The norovirus season was considered to start in calendar week 27 and end in calendar week 26 of the following year

^b^ NHS 111 data runs from 2014/15 to 2018/19

^c^ GP data runs from 2012/13 to 2018/19

**Fig. 1:**
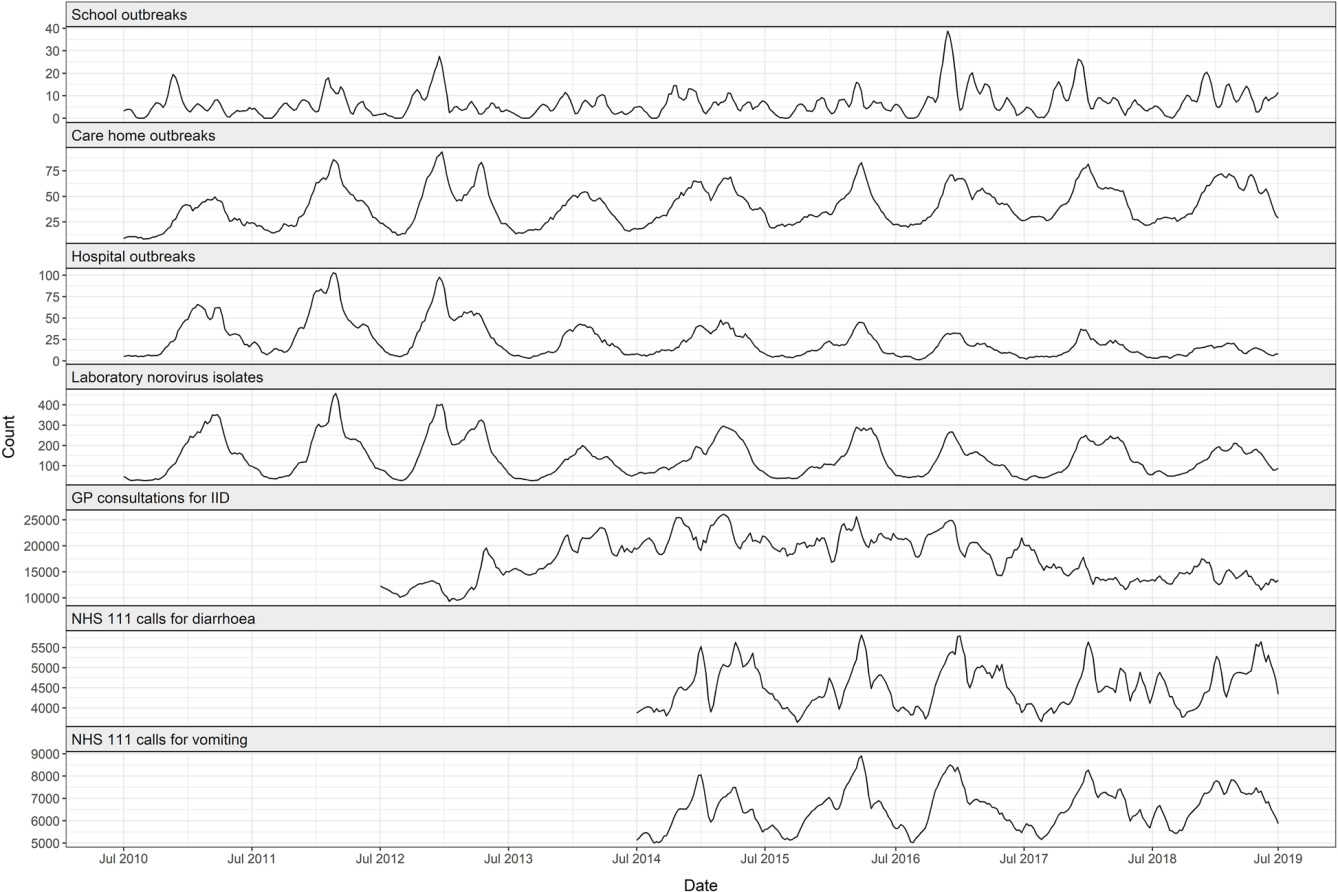
Time trends in surveillance datasets, based on a 4-week rolling average

Figure 1 shows the time trends of each surveillance dataset. Laboratory norovirus reports demonstrated a distinct seasonal trend with a peak during the winter and spring each year, although the exact timing of the peak varied. Hospital and care home outbreaks closely mirrored the seasonality of laboratory norovirus reports, but school outbreaks showed more variability. There were visible peaks in school outbreaks coinciding with laboratory reports in six of the surveillance years, but less defined peaks in the remaining three years. Winter/spring peaks were also captured in NHS 111 data for both vomiting and diarrhoea but GP consultations for IID showed a less clear seasonal trend.

**Fig. 2:**
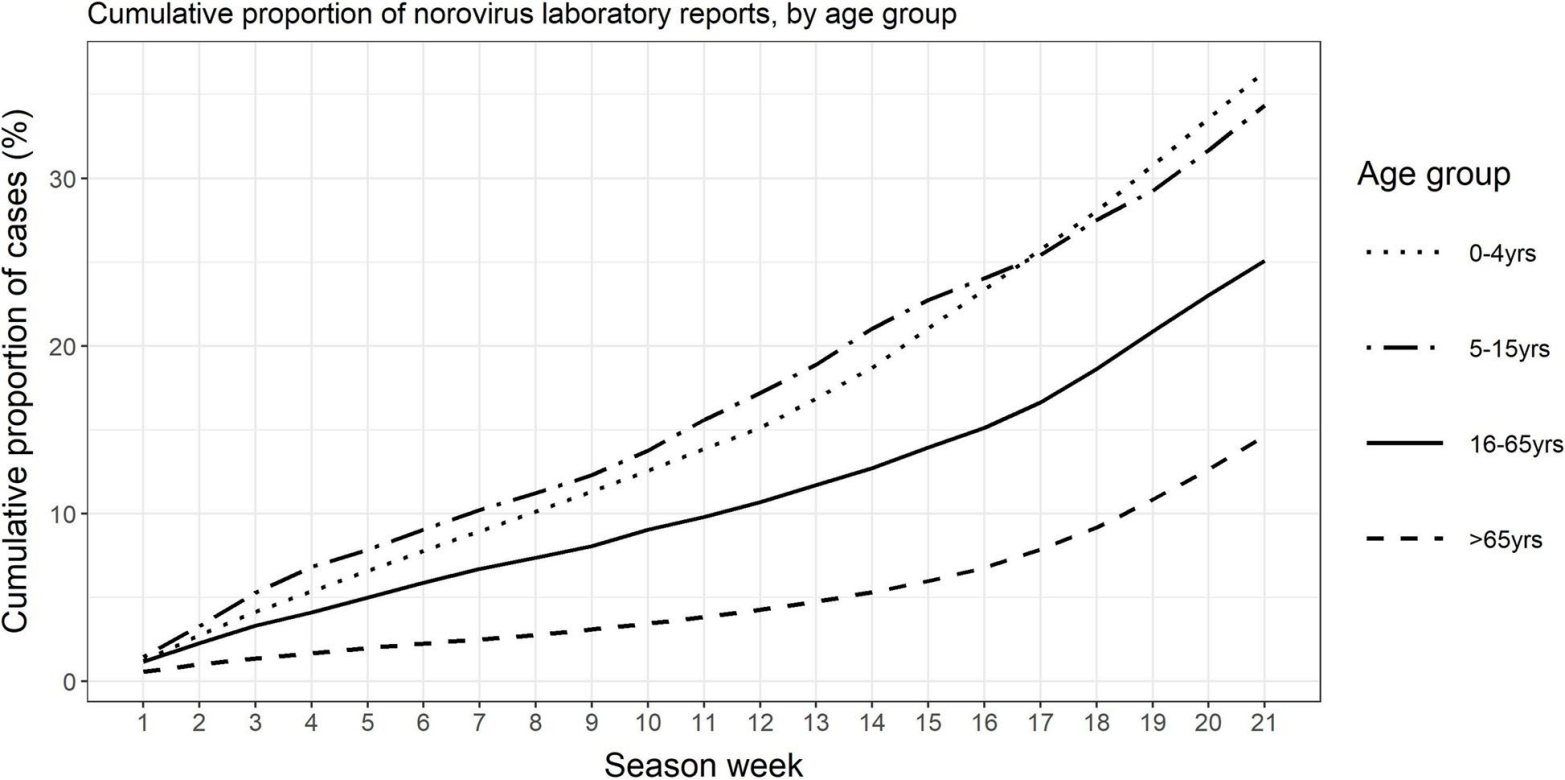
Cumulative proportion of norovirus laboratory reports (2010/11–2018/19), by age group *Season week 1 corresponds to ISOweek 27

Based on the median season week of reported cases and outbreaks, school outbreaks occurred earlier in the norovirus season than the other surveillance indicators (week 25), two weeks earlier than NHS 111 calls and GP consultations, and 4–5 weeks earlier than care home and hospital outbreaks (Table 1). Laboratory reports had the latest median season week (week 32), seven weeks after school outbreaks. Whilst GP consultations and NHS 111 calls in children did not have an earlier median season week than adults, laboratory reports in children occurred 4 weeks earlier than for adults. Further analysis of laboratory samples by age showed that cases of laboratory-confirmed norovirus in children started increasing earlier in the season than cases in adults (Fig. 2). In preschool (< 5yrs) and school-aged children (5-15yrs), 25% of cases were reported by week 17, compared to week 21 in adults (16-65ys) and week 25 in elderly (> 65yrs).

### Correlation analysis

As shown in Table 2, laboratory-confirmed cases of norovirus in children showed a positive correlation with cases in adults and provided a 1–2-week lead time across the norovirus season (r_s_ 0.80, *p* < 0.001). Children provided a 1-week lead time ahead of adults for both NHS 111 vomiting calls (r_s_ 0.78, *p* < 0.001) and NHS 111 diarrhoea calls (r_s_ 0.69, *p* < 0.001). GP consultations for children did not appear to be correlated with consultations for adults,with no evidence of significant lead or lag times.

**Table 2:** Spearman’s rank correlation, showing the relative temporal position of cases in children (0-15yrs) in relation to adults (≥ 16yrs) (by week)

Surveillance dataset	Lead time					Lag time			
	+ 4 weeks	+ 3 weeks	+ 2 weeks	+ 1 week	Concurrent	-1 week	-2 weeks	-3 weeks	-4 weeks
Laboratory norovirus reports	0.75	0.78	0.80	0.80	0.75	0.67	0.58	0.48	0.40
NHS 111 calls for diarrhoea^c^	0.68	0.66	0.64	0.69	0.64	0.39	0.20	0.10	0.02
NHS 111 calls for vomiting^c^	0.62	0.71	0.76	0.78	0.73	0.58	0.48	0.41	0.37
GP consultations^ab^	-0.09	-0.09	-0.30	-0.21	0.14	-0.03	-0.15	-0.01	-0.09

^a^ Age groups for GP consultations are 0-14yrs and ≥ 15yrs

^b^ GP data runs from 2012/13 to 2018/19

^c^ NHS 111 data runs from 2014/15 to 2018/19

When controlled for school holidays, school outbreaks were positively correlated with outbreaks in care home and hospitals and provided a 3-week lead time ahead of outbreaks in both settings (r_s_ 0.76, *p* < 0.001 and r_s_ 0.77, *p* < 0.001 respectively) (Table 3). School outbreaks also provided a 3-week lead time ahead of laboratory surveillance data (r_s_ 0.69, *p* < 0.001) and NHS 111 calls for diarrhoea (r_s_ 0.59, *p* < 0.001), as well as a 2-week lead time ahead of NHS 111 calls for vomiting (r_s_ 0.80, *p* < 0.001). GP consultations were concurrent with outbreaks in schools (r_s_ 0.55, *p* < 0.001).

**Table 3:** Spearman’s rank partial correlation, comparing outbreaks in schools in relation to listed surveillance indicator, controlled for school holidays

Relative temporal position of school outbreaks in relation to listed dataset	Lead time					Lag time			
	+ 4 weeks	+ 3 weeks	+ 2 weeks	+ 1 week	Concurrent	-1 week	-2 weeks	-3 weeks	-4 weeks
Care home outbreaks	0.73	0.76	0.73	0.68	0.61	0.46	0.42	0.33	0.25
Hospital outbreaks	0.73	0.77	0.72	0.67	0.61	0.50	0.41	0.34	0.30
Laboratory norovirus reports	0.68	0.69	0.64	0.59	0.52	0.43	0.35	0.23	0.16
NHS 111 calls for diarrhoea^c^	0.54	0.59	0.55	0.34	0.22	0.06	0.00	-0.02	-0.10
NHS 111 calls for vomiting^c^	0.63	0.76	0.80	0.69	0.60	0.48	0.41	0.38	0.30
GP consultations^ab^	0.16	0.24	0.24	0.52	0.55	0.48	0.37	0.21	0.23

^a^ Age groups for GP consultations are 0-14yrs and ≥ 15yrs

^b^ GP data runs from 2012/13 to 2018/19

^c^ NHS 111 data runs from 2014/15 to 2018/19

### Breakpoint analysis

When laboratory reports, GP consultations and NHS 111 calls were broken down into child and adult age groups, the breakpoint analysis identified an earlier increase in laboratory reports in children in all nine surveillance years, 3–10 weeks ahead of an increase in adult cases (Table 4). GP consultations and NHS 111 calls for children also led adults, with breakpoints occurring earlier in all five seasons for NHS 111 calls, and five out of six seasons for GP consultations. There was an earlier increase in school outbreaks compared to other surveillance indicators in five out of nine surveillance years (Table 5). No peak was identified for school outbreaks in two of the years, and the breakpoint was concurrent or lagged behind other measures in the remaining two years.

**Table 4:** Season week of first detected breakpoint with 95% confidence intervals, based on 4-week rolling average, by norovirus season and age group

Surveillance dataset	2010/11	2011/12	2012/13	2013/14	2014/15	2015/16	2016/17	2017/18	2018/19
***Laboratory norovirus reports***									
Children	16 (14–17)	16 (15–17)	13 (11–14)	16 (15–17)	9 (5–13)	15 (14–18)	14 (12–15)	14 (12–15)	14 (5–21)
Adults	24 (23–25)	24 (23–25)	17 (16–18)	22 (20–25)	19 (17–20)	18 (16–23)	18 (17–19)	20 (19–21)	21 (18–23)
***GP consultations for IID***									
Children	NA	NA	13 (6–14)	19 (18–20)	14 (11–15)	15 (11–18)	13 (11–14)	-	13 (49–18)
Adults	NA	NA	41 (40–42)	21 (19–22)	27 (20–41)	29 (23–35)	48 (46–50)	-	9 (8–11)
***NHS 111 calls for diarrhoea***									
Children	NA	NA	NA	NA	15 (12–16)	17 (15–20)	15 (14–16)	15 (13–17)	19 (17–20)
Adults	NA	NA	NA	NA	24 (19–25)	34 (31–37)	17 (12–18)	23 (17–24)	24 (21–25)
***NHS 111 calls for vomiting***									
Children	NA	NA	NA	NA	15 (13–16)	16 (15–18)	15 (14–16)	15 (14–16)	17 (15–19)
Adults	NA	NA	NA	NA	23 (18–24)	25 (22–26)	17 (13–18)	23 (19–24)	24 (21–25)

**Table 5:** Season week of first detected breakpoint with 95% confidence intervals, by norovirus season, based on a 4-week rolling average

Surveillance dataset	2010/11	2011/12	2012/13	2013/14	2014/15	2015/16	2016/17	2017/18	2018/19
School outbreaks	13 (8–14)	27 (23–28)	13 (10–14)	-	13 (11–15)	-	18 (11–19)	12 (9–13)	13 (4–16)
Care home outbreaks	21 (20–22)	24 (23–25)	16 (15–17)	20 (19–21)	15 (14–16)	30 (28–31)	18 (17–19)	19 (17–20)	20 (19–21)
Hospital outbreaks	22 (21–23)	23 (22–24)	17 (15–18)	22 (21–23)	21 (20–22)	21 (20–24)	17 (15–18)	20 (18–21)	17 (13–20)
Laboratory norovirus reports	24 (23–25)	24 (23–25)	17 (15–18)	22 (21–24)	18 (16–19)	18 (16–22)	17 (15–18)	20 (19–21)	20 (17–22)
GP consultations for IID	NA	NA	40 (38–41)	21 (20–22)	14 (11–15)	29 (24–33)	13 (11–16)	-	11 (9–12)
NHS 111 calls for diarrhoea	NA	NA	NA	NA	16 (11–17)	33 (30–34)	16 (15–17)	21 (17–22)	24 (22–25)
NHS 111 calls for vomiting	NA	NA	NA	NA	15 (13–16)	17 (16–18)	15 (14–16)	19 (18–20)	20 (19–21)

## Discussion

Whilst previous studies have demonstrated an important role for children in the transmission of norovirus infection [27–30], it was uncertain whether or not children were affected earlier in the norovirus season than adults. This study found that outbreaks of IID in schools had an earlier median season week than outbreaks in other settings and correlated well with outbreaks in care homes and hospitals, laboratory norovirus reports and NHS 111 calls for vomiting. School outbreaks occurred 3-weeks before care home and hospital outbreaks, norovirus laboratory reports and NHS 111 calls for diarrhoea, and provided a 2-week lead time ahead of NHS 111 calls for vomiting. Children provided a lead time ahead of adults for both norovirus laboratory reports (+ 1–2 weeks), NHS 111 calls for vomiting (+ 1 week) and NHS 111 calls for diarrhoea (+ 1 week) but occurred concurrently with adults for GP consultations. Breakpoint analysis revealed an earlier seasonal increase in cases in children compared to adults for laboratory, GP and NHS 111 data, with school outbreaks increasing earlier than other surveillance indicators in five out of nine surveillance years.

Our study supports the findings of Bernard et al. who identified that laboratory-confirmed norovirus cases in Germany started rising in children earlier in the season than adults and elderly [25]. However, in our study laboratory reports still had the latest median season week of all the surveillance datasets. Previous studies had identified that telehealth calls for vomiting provided a lead time ahead of laboratory surveillance data, with vomiting calls for young children providing the earliest indication of norovirus season [41, 42]. In this study, whilst NHS 111 calls for vomiting did have an earlier median season week than laboratory reports, school outbreaks had the earliest median season week, demonstrating a lead time ahead of other surveillance indicators and an earlier seasonal increase in five out of nine surveillance years. This would suggest a potential role for school outbreak data in the surveillance of norovirus which could provide an earlier warning of the start of norovirus season compared to existing indicators. Studies have previously explored the role of other school-based surveillance systems, such as those based on school absenteeism, and have found syndrome-specific absences for influenza provided a lead time ahead of traditional surveillance systems during the H1N1 pandemic [43–45]. Whilst school outbreak data were not norovirus specific, high levels of viral shedding and a low infective dose make norovirus a common cause of outbreaks in semi-enclosed settings [15]. In this study, the close mirroring of time trends of outbreaks in care homes and hospitals with laboratory confirmed norovirus cases would suggest that norovirus was driving the majority of outbreaks in these settings. Whilst school outbreaks did not mirror norovirus trends as closely, the correlation with laboratory-confirmed cases would suggest that norovirus was a likely cause of many of the IID outbreaks reported in schools. The utility of outbreak data for norovirus surveillance may be further improved by enhancing sampling and laboratory testing in community outbreaks to confirm norovirus as the causative organism.

Within individual surveillance datasets, the breakpoint analysis suggested children provided an earlier signal than adults across all datasets and for all norovirus seasons, a finding that was less consistent in the correlation analysis and not reflected in the descriptive analysis. The correlation analysis identified a lead time for children ahead of adults in laboratory data and NHS 111 calls for both vomiting and diarrhoea, consistent with findings that telehealth calls for vomiting in young children provide an earlier signal than vomiting calls for all ages combined [42]. However, no lead time was identified for GP consultations and the correlation coefficients suggested no correlation between trends in children and those in adults. A possible explanation for this finding is that surveillance indicators which are based on broad syndromic definitions will also capture causes of IID other than norovirus. Consequently, children and adults may exhibit different trends in GP consultations, caused by different organisms. This could affect the application of this study’s findings to other settings, as seasonal trends in IID may be driven by different organisms in other countries. This is particularly pertinent to rotavirus, where vaccine coverage will impact on the relative importance and burden of this pathogen amongst children and consequently the seasonal trends of IID observed in this age group.

However, different syndromic indicators may be better at capturing certain pathogens than others. As most norovirus infections are mild and short-lived, people with norovirus may be less likely to require a GP consultation and longitudinal data suggest there are 23 norovirus cases in the community for every one which presents to the GP [5]. GP data may, therefore, be better at detecting trends in organisms which cause more severe or prolonged symptoms. This could explain why the GP data in this study did not reflect the seasonal trends seen in the other datasets. For NHS 111 data, whilst both diarrhoea and vomiting are features of norovirus infection, there is evidence that vomiting may be a more prevalent feature amongst children and diarrhoea more common amongst adults [27, 46]. This could make calls for vomiting a more sensitive indicator of norovirus infection amongst children and may explain why school outbreaks correlated better with NHS 111 vomiting calls than diarrhoea calls (r_s_ 0.80 and r_s_ 0.59 respectively). This highlights the challenge of using syndrome-based surveillance data to monitor specific organisms in the community and it should be considered that the utility of different syndromic surveillance indicators may alter depending on the organism and age group.

### Strengths and limitations

This study utilises national surveillance data on over 65 000 laboratory confirmed cases of norovirus, 33 000 outbreaks of IID and over 9 million calls and consultations for IID across nine norovirus seasons. The use of routine surveillance data for this study allows large numbers of cases to be captured across multiple norovirus seasons. However, all surveillance data is subject to reporting bias, as only cases which present to healthcare will be captured in the datasets. This also applies to outbreaks, which are voluntarily reported to Public Health England. Consequently, it cannot be determined whether the lack of a peak in school outbreaks in certain years is the result of fewer outbreaks occurring or lower levels of reporting from schools. Equally, differences in reporting behaviour between children and adults will also be reflected in the data, although as reporting biases are unlikely to change throughout a given norovirus season, it is more likely to affect overall case numbers rather than trend.

An additional limitation of using school-based data are the natural breaks in data collection which occur during school holidays. This could affect the utility of school outbreaks as a surveillance indicator for norovirus. It is well documented that school holidays impact on social mixing patterns [47] and there is evidence that the timing of school holidays can impact on transmission and the size of peaks for other infectious diseases, such as influenza [38, 48, 49]. In this study, in the years where the breakpoint analysis did not identify a seasonal peak in school outbreaks, norovirus laboratory reports increased later in the season and peaked after the school Christmas break. The same occurred for the year where school outbreaks had a later breakpoint than other surveillance datasets. Consequently, the timing of school holidays relative to the norovirus peak may be affecting the size and timing of peaks in school outbreak data. This could affect the potential of school outbreak data to provide an early warning ahead of other surveillance indicators in any given year.

## Conclusion

Children are recognised as important transmitters of norovirus infection and this study explored whether cases in children and outbreaks in schools occurred earlier in the norovirus season than cases and outbreaks amongst adult age groups. Trends in school outbreaks had a lead time ahead of other surveillance indicators and cases in children provided a lead time ahead of adults for norovirus laboratory reports and NHS 111 calls for both vomiting and diarrhoea. Cases in children started increasing earlier in the season than adults for all surveillance datasets across the study period and school outbreaks increased earlier than other surveillance indicators in five out of nine surveillance years. These findings suggest that monitoring cases and outbreaks of norovirus in children could provide an early warning of seasonal norovirus infection. However, the utility of using school outbreaks as a surveillance indicator may be affected by the timing of school holidays in relation to the norovirus peak in any given year. Furthermore, both school outbreak data and cases in children from syndromic surveillance are not norovirus specific and hence will also capture other causes of IID. The use of school outbreak data as an early warning surveillance indicator may be improved by enhancing sampling in community outbreaks to confirm the causative organism.

## Data Availability

The data that support the findings of this study are available from the UK Health Security Agency (UKHSA) but restrictions apply to the availability of these data and they are not publicly available. Data are, however, available from the authors upon reasonable request and with permission of UKHSA.

## Abbreviations

GP: General practice
HNORS: Hospital Norovirus Outbreak Reporting System
IID: Infectious intestinal disease
NHS: National Health Service
PHE: Public Health England
